# Is There a Place for Immunotherapy for Metastatic Microsatellite Stable Colorectal Cancer?

**DOI:** 10.3389/fimmu.2019.01816

**Published:** 2019-08-06

**Authors:** François Ghiringhelli, Jean-David Fumet

**Affiliations:** Department of Medical Oncology, Centre Georges François Leclerc, Dijon, France

**Keywords:** colorectal cancer, checkpoint inhibitor, mismatch repair deficiency, combination therapy, PD-1, PD-L1

## Abstract

Immunotherapy using checkpoint inhibitor targeting PD-1 and PD-L1 revolutionized the treatment of microsatellite instable metastatic colon cancer. Such treatment is now a standard of care for these patients. However, when used as monotherapy checkpoint inhibitors targeting PD-1 and PD-L1 are not effective in metastatic colorectal cancer patients with microsatellite stable tumors. Recent advances in biology provide a rationale for this intrinsic resistance and support the evaluation of combination therapy to reverse resistance. This article will highlight recent findings on the mechanism of intrinsic resistance and recent advances in clinical trials for combination therapy.

## Introduction

Tumor microenvironment (TME) plays an important role in cancer progression and in the response to therapy. Increasing data in the literature underlines that CD8 T cells and tumor-infiltrating lymphocyte (TIL) accumulation in the tumor bed are biomarkers of good outcome in most types of cancers (1). In the context of colorectal cancer, the presence of CD8 T cells in the tumor bed and invasive margin is strongly associated with outcome. Jerome Galon's team's publications have shown that time to recurrence and overall survival strongly correlate with the strength of the *in-situ* adaptive immune reaction in the colon tumor core and invasive margin (2, 3). They proposed that solid tumors' intra-tumoral immune context (i.e., type, functional orientation, density, and location of immune cells) could be a dominant determinant of clinical outcome (4). These data underline that colorectal cancers are frequently widely invaded by immune cells and suggest that immunotherapy could be a suitable therapy for such patients. Based on this observation, anti PD-1 mAb was tested in advanced metastatic colorectal cancers. However, initial reports of phase I trials were very disappointing, with only 1 of 33 patients with colorectal cancer with objective clinical response to this treatment (5, 6). Importantly, the responding patient differed from others due to the mismatch-repair deficiency (dMMR). dMMR is a small fraction of whole colorectal cancer. dMMR status is due to a mutation in genes involved in DNA mismatch repair (MLH1, MSH2, MSH6, PMS2, EPCAM). Such mutations can be exclusively somatic or constitutional, in the context of Lynch syndrome. These tumors represent around 15% of localized colorectal tumors and about 3–4% of metastatic colon cancers (7). Recently, Le et al. reported a phase 2 clinical trial to evaluate the efficacy of pembrolizumab, an anti PD-1 immune checkpoint blocker, in colorectal cancer patients with either dMMR or proficient MMR (pMMR status). In this trial, only treatment-refractory metastatic colon cancer patients were included. Objective response was 40% in patients with dMMR tumors, while no patient had an objective response in the pMMR group. The median progression-free survival reached 5 months in dMMR patients but only 2 months in pMMR patients (8). Such data support that checkpoint inhibitors targeting PD-1 are only effective in dMMR tumors. In this review, we will explain why dMMR tumors are sensitive to checkpoint inhibitors and we will study the different mechanisms of pMMR tumors' intrinsic resistance and how to circumvent them.

## RationalE OF Checkpoint Inhibitors' Efficacy in Microsatelite Instable Tumors

dMMR status relies on epigenetic silencing or mutations in DNA mismatch repair genes (9, 10). This anomaly induces genetic aberrations due to DNA replication errors in microsatellites, short tandemly repeated DNA sequences. Such an anomaly is called microsatellite instability (9) and is classically diagnosed by the variable length of DNA microsatellites, some mononucleotide and dinucleotide repeats. dMMR mutations induce accumulation of DNA replication errors in both coding and non-coding DNA regions, which can be point or frameshift mutations (9). This mechanism induces mutation accumulation at a 10- to 50-fold higher rate than in pMMR tumors. The inactivation of MMR increased the mutational burden and led to dynamic mutational profiles, which resulted in the persistent generation of neoantigens, whereas MMR-proficient cells exhibited stable mutational load and neoantigen profiles over time (11). Consequently, when present in the coding sequence such mutations induce the generation of a large number of neoantigens, which could be presented as neoantigenic peptides by HLA molecules of both tumor and antigen presenting cells and be recognized as foreign antigens by T cells (12). Such a mechanism could explain why dMMR tumors present higher CD8 cytotoxic T and Th1 helper cells infiltration, resulting in a better prognosis when tumors are non-metastatic (10). Mutant neoantigens are recognized by tumor-antigen-specific T cells, present in growing tumors, and able to limit both tumor growth and metastatic process. In experimental settings, these CD8 T cells can be reactivated following treatment with anti-PD-1/anti-CTLA-4 and mediate tumor rejection (13). So, we can hypothesize that a high level of neoantigens in localized tumor dMMR tumors might explain their better prognosis via a more robust immunoediting. In the metastatic setting, we could hypothesize that CD8 and Th1 infiltrating dMMR tumors are exhausted and could be reactivated by checkpoint inhibitors (14).

**Figure 1:**
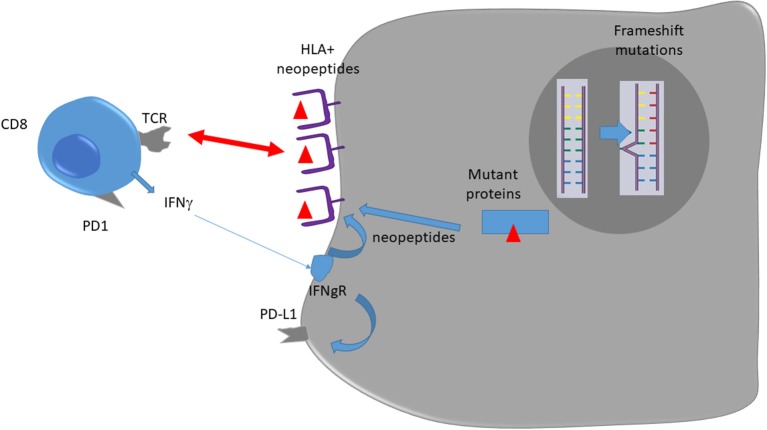
Immune response against dMMR tumors.

In dMMR tumors, CD8 and Th1 express high levels of multiple checkpoints inhibitors such as programmed death-1 (PD-1), cytotoxic T lymphocyte–associated antigen 4 (CTLA4), and lymphocyte activation gene 3 (LAG3) in comparison to pMMR tumors (15). These markers underline that intratumoral T cells present an exhausted status. Exhausted CD8 T cells are T cells that emerge during chronic antigen stimulation. These cells are initially effector cells, which produce a high level of cytotoxic molecules and interferon gamma (IFNγ). In the absence of complete tumor eradication, the sustained antigen stimulation restrains T cells' capacity to produce cytotoxic molecules and inflammatory cytokines such as IFNγ (16). In addition, dMMR colorectal cancer (CRC) may present an increased expression of tumor PD-L1, which has been correlated with checkpoint inhibitor efficacy in different tumor types in a retrospective study (17). Such data might explain both checkpoint inhibitors' efficacy in such tumors and absence of spontaneous tumor eradication due to T cell exhaustion [(12); Figure 1].

## Mechanism of Intrinsic Resistance in Microsatelite Stable Tumors

### Immunoexclusion

Immunohistological analysis of colon cancer revealed that CD8 infiltration is mainly located in the invasive margin around tumors (18). Among dMMR tumors, T cells were more abundant at the invasive margin than in the tumor core, thus suggesting that a mechanism of immunoexclusion could be involved in the absence of T cells in the tumor core. Absence of T cells in the tumor core may blunt the efficacy of checkpoint inhibitors (19).

Some experimental data on mice preclinical models of pMMR colon cancer summarize this phenomenon. Transforming growth factor–β (TGFβ), an immunosuppressive cytokine associated with bad prognosis, was observed in the tumor bed of preclinical models of colon cancer (20, 21). TGFβ acts on the fibroblastic stroma, increases fibrosis and limits tumor core T cell invasion. Inhibition of TGFβ using a pharmacological inhibitor or a mAb promotes T cell recruitment to the tumor bed and efficacy of checkpoint inhibitors (22). Recently bifunctional checkpoint inhibitor, the fusion protein M7824, comprising the extracellular domain of human TGFβRII linked to the C-terminus of human anti-PD-L1, was developed and showed important efficacy in preclinical models. M7824 treatment promoted CD8+ T cell and NK cell activation, and both of these immune populations were required for optimal M7824-mediated tumor control. M7824 was superior to TGFβ- or αPD-L1-targeted therapies when in combination with a therapeutic cancer vaccine (23).

Immunoexclusion could also be related to tumor cells' intrinsic mechanism. pMMR tumors are characterized by the presence of the activation of WNT/β-catenin signaling. In contrast, this pathway is rarely activated in dMMR colon tumors (24). Previous data obtained in melanoma underline that WNT/β-catenin signaling activation is involved in the mechanism of immune exclusion. WNT/β-catenin signaling induces transcriptional repression of chemokine genes such as CCL4, essential for intratumoral homing of dendritic cells to the tumor bed. In particular, CCL4 expression induces recruitment of Batf3 positive dendritic cells which are essential for T cell priming, activation and recruitment to the tumor site (25, 26). Activation of tumor-intrinsic WNT/β-catenin signaling was also tested in TCGA pan cancer data (27). This analysis across 31 tumors determined that 28 (90%), including colon cancer, showed activated β-catenin signaling in the non-T cell-inflamed subset, demonstrating this observation is relevant in most cancer types. Targeting WNT/β-catenin could be a strategy to improve immunotherapy efficacy (28).

### Lack of Antigens

To induce an antitumor immune response, tumor cells must contain antigens detected by cytotoxic T cells. pMMR tumors have fewer mutations than dMMR tumors. Recent literature shows that there is a strong association between mutation presence and response rate to checkpoint inhibitors used as monotherapy (29). The number of non-synonymous mutations is called the tumor mutational burden (TMB). The median TMB of a pMMR tumor is 4 mutations/MB, which classifies this tumor as a low TMB tumor. In comparison, TMB mean of dMMR tumor is 30 mutations/MB (30). However, the median number of mutations in pMMR is similar to the one found in ovarian cancer or hepatocellular carcinoma, which present some response to checkpoint inhibitor used as monotherapy. Such data suggest that additional mechanisms other than TMB explain resistance to checkpoint inhibitors in pMMR tumors.

Despite the lack of antigens in pMMR tumors most colon cancer tumors are infiltrated by CD8 T cells. There is evidence that tumor-specific T cells targeting neoantigens play a role in tumor control (13, 14, 29, 31), but in most tumor types antigen specificities are unknown. A hypothesis to explain the lack of efficacy could be that CD8 tumor infiltrating cells are non-tumor specific cells and classify as bystander cells. In a recent Nature paper (29), the authors studied the antigen specificity of CD8 tumor infiltrated cells in human lung and colorectal cancer. They observed that only very few CD8^+^ TILs are specific for tumor antigens. Most TILs recognize a wide range of infectious epitopes such as Epstein–Barr virus, cytomegalovirus or influenza virus. Similarly, specific T cell response was tested in another report concerning melanoma, colon cancer and ovarian cancer (32). While in melanoma tumors specific T cells represent 60% of tumor infiltrated CD8, in ovarian cancer and colon cancer they represent only 5 and 9%, respectively. Such data underlines that only a minority of CD8 TILs recognize tumor antigens in pMMR tumors, therefore a lack of antigen specificity may at least partly explain resistance to immunotherapy.

Activation of the mitogen activated protein kinase (MAPK) pathway is found in around 60% of pMMR colon cancers due to a constitutive activation of the small GTPase K-Ras (Kirsten rat sarcoma viral oncogene homolog) or other N-RAS (neuroblastoma rat sarcoma viral oncogene homolog). Such mutations are more frequent in pMMR than in dMMR tumors and lead to a constitutive activation of the downstream pathway effectors molecules MEK (Mitogen/Extracellular signal regulated Kinase) and ERK1 and/or ERK2 (33, 34). Activation of the MAPK pathway reduces MHC class I molecule expression on tumor cells of different cancer types such as melanoma, breast cancer and colon cancer (35–37). Pre-clinical experiments showed that MAPK inhibition, using MEK inhibitors, resulted in MHC class I molecules upregulation in tumor cells and increased CD8 T infiltration in tumor core (38). Such data provide a rationale to combine MEK inhibitors and checkpoint inhibitors in RAS mutated tumors to enhance MHC class I molecule expression and to enhance tumor recognition by infiltrated CD8 T cells (39). Based on these results a phase I with cobimetinib and atezolizumab was started and confirmed biological activity of this combination in CD8 T cells recruitment and induction of HLA expression (40). Subsequently, a phase III trial was then started in patients with pMMR advanced treatment-refractory colorectal cancer and compared the combination of cobimetinib and atezolizumab with atezolizumab alone or regorafenib (41). Neither atezolizumab monotherapy nor combination atezolizumab and cobimetinib demonstrated significantly improved OS compared to regorafenib.

## Immunosuppression

In addition to effector populations, like CD8 T cells and antigen presenting cells, the presence of immunosuppressive cells may control antitumor immune response and could blunt the efficacy of checkpoint inhibitors. The two main immunosuppressive cells are FOXP3 regulatory T cells (Tregs) and the myeloid derived suppressor cells (MDSC).

Tregs have the capacity to inhibit most immune cells. These cells accumulate during tumor growth and are frequently associated with poor prognosis in various types of cancers (42–46). However, in colorectal cancers their role is complex. Indeed, some studies looking at FOXP3 positive cell accumulation in colorectal tumors suggest that a better prognosis is associated with the presence of such infiltrates (30, 45–48). This event probably relies on the fact that T cell infiltration is a strong surrogate marker of good prognosis in colorectal cancer and that Foxp3 accumulation is strongly correlated with accumulation of other immune cells (15, 49). The role of Treg infiltration in colorectal cancer became even more complex with the discovery of two types of Tregs in colon cancer. These two types of cells could be differentiated by their level of Foxp3 expression (low vs. high) (50). Only FOXP3 high cells are immunosuppressive and their accumulation in colon cancer is a surrogate marker of poor prognosis. In contrast, Foxp3 low non-suppressive Treg cells are not a factor of bad prognosis. These cells are associated with the presence of *Fusobacterium nucleatum* which is also associated with dMMR status (51). Together, such data raise the hypothesis that dMMR tumors are infiltrated with Foxp3 low non-suppressive Tregs, which are recruited due to the presence of *Fusobacterium nucleatum* and also probably due to other chemoattractant agents, while pMMR tumors are mainly invaded by Foxp3 high immunosuppressive Tregs which blunt immune response. Depletion of FOXP3 high Treg cells from tumor tissues may augment antitumor immunity and should be tested in combination with checkpoint inhibitors in pMMR tumors.

Myeloid-derived suppressor cells (MDSC) are a heterogeneous population of myeloid cells with monocytic and neutrophilic phenotypes. These cells are blocked at immature stages of differentiation and exert an immunosuppressive role in both innate and adaptive immune cells. These cells are absent in healthy humans but accumulate in blood, lymph nodes, bone marrow, and tumors during cancer growth (52, 53). The accumulation of MDSC was tested in colon cancer and a high level of MDSC was found in the blood of patients with metastatic colorectal cancers (54). This MDSC accumulation is associated with poor prognosis. Preclinical data underline that MDSC elimination could induce CD8 T cell accumulation and reactivation at the tumor site (55), thus suggesting that elimination of such immunosuppressive cells could enhance the efficacy of checkpoint inhibitors.

Secondary immunosuppression due to induction of checkpoint inhibitor expression might also be relevant. We recently reported higher expression of immune checkpoints in dMMR tumors in comparison to pMMR tumors. Immune checkpoint expression is associated with intrinsic poor prognosis in dMMR tumors while its expression does not have an impact on pMMR tumor prognosis (15). Such data suggest that immune checkpoints may be clinically more relevant in dMMR tumors, providing a rationale for a better efficacy of these therapies in this category of colorectal cancer.

## Role of Angiogenesis

Neoangiogenesis has a major role during tumor development. Several oncogenic pathways lead to the production of Vascular Endothelial Growth Factor (VEGF), the main proangiogenic factor during cancer growth (56–58). VEGF acts as a specific proliferating agent for endothelial cells through interaction with its specific receptors, VEGFR1 and R2. Both VEGF and its receptors are expressed at high levels in human colon carcinomas and in tumor associated endothelial cells (59–61). However, few data compare angiogenesis in dMMR and pMMR colorectal cancer. A recent biological study (62) tested the presence in the blood of healthy volunteers and patients bearing metastatic dMMR or pMMR colorectal cancers of endothelial progenitor cells and VEGF. Both parameters were increased in patients with dMMR tumors, suggesting a more important dependency of these tumors to angiogenesis.

VEGF is known to have an important and deleterious effect on the immune system. Notably, VEGF could blunt dendritic maturation through STAT3 induction in myeloid cells. VEGF is also known to affect immunosuppression. VEGF could promote MDSC accumulation (63). In patients with cancer, a correlation between disease stage, VEGF-A levels and MDSC accumulation was observed (64, 65). This accumulation is related to the positive effect of VEGFR2 on STAT3 activation, which induces expansion and activation of MDSCs (66, 67). VEGF could also promote Treg cell expansion. In particular, dendritic cell and MDSC activation by VEGF induces IL-10 and TGF-β. These events promote Treg cell expansion (66, 68, 69). VEGF-A could also directly induce Treg proliferation due to their high expression level of VEGFR2 (70). Finally, VEGF is also produced by type 2 tumor-associated macrophages (TAM), which accumulate in colorectal cancer and are associated with poor prognosis (71–74).

VEGF production in the tumor bed induces pathological vascularization which could lead to insufficient vascularization and hypoxia. Such hypoxia is well-known to impede CD8 TILs-mediated lysis of tumor cells (75). VEGF-A neutralization induces tumor vasculature normalization and restores CD8 TILs' effector functions. Taken together, these results show a direct link between tumor vasculature normalization and enhanced immune cell infiltration.

**Figure 2:**
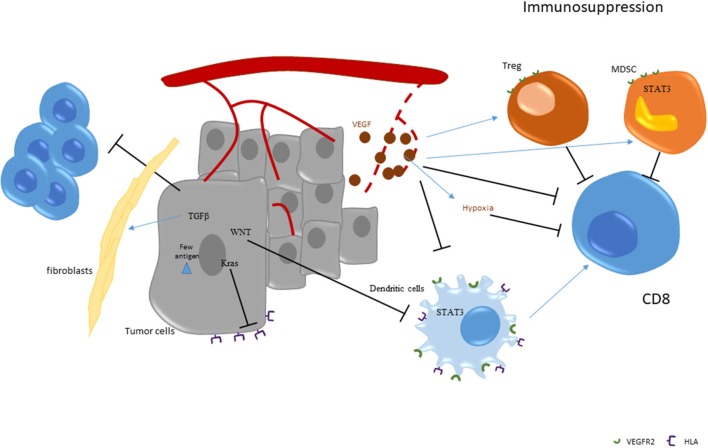
Strategies of immunoescape in pMMR tumors.

The above-described data provide a rationale to use anti-VEGF therapies to limit immunosuppression and to restore an effective antitumor immune response. In mice, anti-VEGF antibody could enhance dendritic cell maturation, resulting in an increase in number and functions of tumor infiltrating dendritic cells (76). Anti VEGF-A or tyrosine kinase inhibitor like sunitinib also led to a significant reduction of MDSCs in peripheral blood in animal models (77–79). Moreover, anti-VEGF could also decrease Treg accumulation due to a direct effect on VEGFR2 and an indirect effect on dendritic cells and MDSCs (70). In patients with colorectal cancer, treatment with chemotherapy and bevacizumab was shown to decrease Treg accumulation and proliferation [(70); Figure 2].

## Hope, Success, and Failure of Current Clinical Trials for pMMR Colorectal Cancer

First, clinical trials in patients with advanced disease show that monotherapy with anti PD-1 is not effective in pMMR colon cancer. The seminal report from Le et al. observed no RECIST objective response in pMMR patients. Nevertheless, two patients with stable disease were observed and a progression-free survival rate of 11% at 20 weeks was found (2 of 18 patients; 95% CI, 1–35) (8). Recently, Chen et al. (80), reported the efficacy of the combination of durvalumab and tremelimumab in heavily pretreated pMMR colorectal cancer in a randomized phase II study. One hundred and twenty patients were included. Patients in an immunotherapy group presented a median OS of 6.6 months vs. 4.1 months in the control arm (0.70; 90% CI (0.53–0.92); *p* = 0.03). In contrast, no difference was observed in terms of progression-free survival. Treatment toxicity was classical for such therapy. This trial suggests that anti-PD-L1 plus anti-CTLA4 combination therapy could have a modest effect in patients previously pretreated for colorectal cancer with a pMMR tumor.

Regorafenib, a potent inhibitor of angiogenic and oncogenic kinases, reduced TAM in tumor models. The combination of regorafenib plus a PD1 exhibited superior tumor growth suppression compared to either treatment alone in murine models. Consequently, a phase 1B study tested the combination of regorafinib and nivolumab in 25 pMMR previously-treated colorectal cancer patients. Regorafenib dose was reduced to 80 mg due to skin toxicities. Objective tumor response was observed in 7 pMMR colon cancer patients given 29% of response rate. The blood immunomonitoring showed a reduction of the FoxP3^hi^CD45RA^−^Tregs fraction at the tumor response (81) NCT03406871.

Chemotherapy could be used to promote immune response via a mechanism called immunogenic cell death (82). Oxaliplatin is known to induce this process. Cancer cells killed by oxaliplatin express calreticulin on the cell surface and release HMGB1, ATP, and Type I interferon. Calreticulin is recognized by dendritic cells which then phagocyte dead bodies. HMGB1 and ATP promote antigen presentation and activation of dendritic cells, resulting in optimal activation of CD8 T cells. Then Type I interferon induces an important recruitment of CD8 to the tumor bed (83). Our group showed that 5-fluorouracil could induce MDSC elimination (55, 84). Therefore, combination of oxaliplatin and 5-Fluorouracil could target both MDSC dependent immunosuppression and activation of effector T cells via immunogenic cell death. However, in preclinical models, FOLFOX regimen was also shown to induce PD-1 expression in CD8 T cells and PD-L1 expression in macrophage and myeloid cells, in a type II interferon dependent manner (85), suggesting that FOLFOX combination with an anti-PD-1 mAb (86) might be useful.

A phase II trial involving 30 patients tested the combination of FOLFOX with pembrolizumab in untreated, unresectable pMMR colorectal cancer. A total of 53% of patients had a RECIST objective response at 24 weeks, with a disease control rate of 100% at 8 weeks (87). Survival data are awaited to determine if such combination is better than FOLFOX alone. Our group also initiated a trial, in first line pMMR patients with RAS mutated colorectal cancer, to test the combination of FOLFOX plus durvalumab and tremelimumab (88). This study is still ongoing.

As with chemotherapy, preclinical data support the capacity of radiotherapy to induce immunogenic cell death and promote activation of antitumoral immune response. However, the radiotherapy schedule may modulate its immune effect. Conventional or hypofractionated radiotherapy induces the release of DNA in the cytoplasm of cancer cells. Such cytoplasmic DNA is recognized by a DNA sensor, STING, which induces Type I IFN production. Without this Type I IFN production, no immune effect of radiotherapy or combination therapy with radiotherapy and checkpoint inhibitor could be observed (89, 90). Surprisingly, a high dose of hypofractionated radiotherapy induced exonuclease TREX1 expression. This exonuclease degrades cytoplasmic DNA and limits Type I IFN production. These data strongly support that the schedule of radiotherapy must be adapted to boost immune response.

In the setting of colorectal cancer, some small trials currently test the combination of checkpoint inhibitors and radiotherapy. A phase II, study test pembrolizumab plus radiotherapy vs. pembrolizumab and surgical ablation of metastases in patients with advanced, refractory pMMR CRC. One partial response in a total of 11 patients was observed in the radiotherapy plus immunotherapy group (91). Many clinical trials are ongoing with external radiotherapy or radioembolization of liver metastases (NCT03104439, NCT03007407, NCT03102047, NCT02837263, NCT03005002, NCT02888743).

Since VEGF has immunosuppressive functions, it can be hypothesized that the use of immunotherapy in combination with anti-VEGF therapies might be useful. Combination of atezolizumab with FOLFOX and bevacizumab was tested in first-line metastatic CRC. This treatment led to a 53% objective response and a median progression-free survival of 14.1 months (92). Survival data are awaited to determine if such combination is better than FOLFOX bevacizumab. Biological data show an induction of cytotoxic T cell signatures and PD-L1 expression as well as CD8+ T-cell accumulation. Based on these data, a maintenance trial called MODUL was initiated in patients with pMMR RAS mutated colorectal cancer. Fist line induction therapy with FOLFOX and bevacizumab was initiated, and if patients had a good response then they were randomized between capacitabine plus bevacizumab with or without atezolizumab maintenance regimen (93). No difference was observed in terms of progression-free survival and overall survival.

Mab targeting EGFR could promote antibody-dependent cytotoxicity and CD8 infiltration as well as antitumor immune response in colorectal cancer (94). Based on these results, association of cetuximab and pembrolizumab was tested in RAS wild-type pMMR colorectal cancer previously pretreated by chemotherapy. In the first treated patients, durable (>16 weeks) disease control was observed in 6/9 patients (95). Trials testing FOLFOX cetuximab plus avelumab in first line (NCT03174405) and nivolumab, ipilimumab with panitumumab in patients with metastatic, refractory, RAS wild-type, pMMR colon cancer (NCT03442569) are ongoing.

## Rationale for new Combination TherapIES

New emerging therapies are currently in development. The first strategy targets the poor antigenicity of pMMR colorectal cancer. Vaccination represents a valuable strategy to artificially induce an antitumor immune response and enhance T cell recruitment to tumor bed. The classical strategy uses shared cancer antigens as tumor vaccines. Vaccines can use whole proteins, specific peptides or whole allogeneic cells. As an example, a vaccine called GVAX was developed for colorectal cancer. This vaccine consists of irradiated allogeneic colon cells modified to express granulocyte-macrophage colony-stimulating factor (GM-CSF). A trial currently tests this therapy with pembrolizumab for advanced pMMR tumors (NCT02981524). An alternative strategy is the usage of a personalized peptide vaccine. Next-generation sequencing on tumor tissue is performed to detect the specific neoantigen of patients' tumors. Then, specific peptides which bind to patient human leukocyte antigen (HLA) and coding for the neoantigen are synthetized. Trials are ongoing to test the combination of this strategy with checkpoint inhibitors (NCT03794128, NCT03480152).

Alternative strategies to enhance immunogenicity could rely on oncolytic vaccines. Such a virus could kill cancer cells and induce a local immune response. Multiple viral platforms are currently under evaluation. Recently, a phase II trial of FOLFOX plus bevacizumab with or without an oncolytic reovirus was performed in RAS mutated colon cancer. An increased response was observed with the virus, but with a shorter median duration of response. Decreased treatment intensity with standard agents occurred and may contributed to the lack of benefit of the virotherapy (96). To induce T cell recruitment, T cell bispecific antibodies could be another solution. An antibody which recognizes both CD3 and a surface tumor antigen induces T cell activation and forces them to detect and kill cancer cells. A drug called TCB-CEA was developed and targets the carcinoembryonic antigen (CEA), which is frequently expressed by colon cancer (97). Evidence of antitumor activity in advanced colorectal cancer was reported in a phase 1 trial which tested CEA-TCB plus atezolizumab (97). Increased intratumoral CD3 T cell infiltration was observed, but some major side effects such as cytokine storm were reported, which raised some caution on the development of this drug.

Another strategy relies on elimination of immunosuppressive cells or molecules. To target MDSC and immunosuppressive macrophages, some inhibitors of CSF1R are currently in development in combination with anti PD-1/PDL1 (i.e., NCT02777710, NCT02452424, NCT02829723, NCT02880371). Some other drugs targeting STAT3 (NCT02851004, NCT03647839) or Bruton's Kinase (NCT03332498) or CCR5 (NCT03631407, NCT03274804) are also in development with anti PD-1/PD-L1 to fight against immunosuppressive myeloid cells. Adenosine is also a major immunosuppressive molecule produced by both MDSC and Tregs. This molecule is generated by CD73 and CD39 molecules which degrade extracellular ATP. Therefore, combination of CD39 or CD73 inhibitors with checkpoints to reduce immunosuppression might be relevant. Clinical trials with anti PD1/PDL1 and anti-CD73 or anti-adenosine receptor are ongoing (NCT02503774, NCT03207867, NCT03549000).

## Conclusion and Future Directions

pMMR tumors are complex for immunotherapy. Despite CD8 T cell infiltration and clear demonstration that CD8 infiltrates are associated with tumor outcome, anti-PD-1 monotherapy is ineffective. Mechanisms such as lack of antigen, RAS, WNT pathway activation and immunosuppression could explain this observation. Recent advances in the understanding of immune responses generated several hypotheses to overcome resistance to checkpoint inhibitors in this pathology. While some disappointing results were observed with MEK inhibitors and antiangiogenic agents, some promising results are observed with radiotherapy or chemotherapy in first line.

New strategies involving vaccination, bispecific mAbs, STAT3 inhibitors and drugs targeting immunosuppression are tested and will probably change the face of pMMR cancer treatments.

## Author Contributions

All authors listed have made a substantial, direct and intellectual contribution to the work, and approved it for publication.

### Conflict of Interest Statement

The authors declare that the research was conducted in the absence of any commercial or financial relationships that could be construed as a potential conflict of interest.
